# Excess suicide attributable to the COVID-19 pandemic and social disparities in South Korea

**DOI:** 10.1038/s41598-022-22751-7

**Published:** 2022-11-01

**Authors:** Jieun Min, Jieun Oh, Soo In Kim, Cinoo Kang, Eunhee Ha, Ho Kim, Whanhee Lee

**Affiliations:** 1 Department of Environmental Medicine, College of Medicine, Ewha Womans University, Seoul, Republic of Korea; 2 Graduate Program in System Health Science and Engineering, College of Medicine, Ewha Womans University, Seoul, Republic of Korea; 3 Department of Public Health Science, Graduate School of Public Health, Seoul National University, Seoul, Republic of Korea; 4 Department of Psychiatry, Ewha Womans University Mokdong Hospital, Ewha Womans University College of Medicine, Seoul, Republic of Korea; 5 Institute of Ewha-SCL for Environmental Health (IESEH), Ewha Womans University College of Medicine, Seoul, Republic of Korea; 6 Institute of Sustainable Development, Seoul National University, Seoul, Republic of Korea; 7 School of Biomedical Convergence Engineering, Pusan National University, Yangsan, Republic of Korea

**Keywords:** Psychology, Risk factors

## Abstract

The impact of COVID-19 pandemic on suicide remains unclear and might differ according to individuals’ socioeconomic characteristics. We aimed to investigate excess suicide attributable to COVID-19 in South Korea, stratified by the outbreak period and individual characteristics. We obtained daily time-series suicide mortality data for January 2017–December 2020 from the Korea National Statistics Office and performed a two-stage interrupted time-series analysis. We estimated excess suicide in 16 regions of Korea using a quasi-Poisson time-series regression model and pooled the region-specific estimates using a mixed-effects multivariate meta-analysis model in the first and second stages, respectively. From February 18 to December 31, 2020, suicide decreased by 9.5% [95% empirical confidence interval (eCI): 3.8%, 15.6%] compared to the number expected from the pre-pandemic period. The decrease in excess suicide risk from the initial pandemic was pronounced during the pandemic’s first and third waves. Further, we found that the decrease in suicide was more evident in individuals who were male [11.7% (95% eCI: 5.5%, 18.0%)], middle-aged [13.7% (95% eCI: 7.8%, 19.6%)], highly educated [12.6% (95% eCI: 6.4%, 19.4%)], and married [13.6% (95% eCI: 8.0%, 20.3%)] than in the general population, based on the point estimates. Our results provide timely evidence to establish public health policies for suicide prevention and suggest the prioritization of resource allocation for mental health of individuals based on individual characteristics.

## Introduction

The coronavirus disease 2019 (COVID-19), caused by severe acute respiratory syndrome coronavirus 2 (SARS-CoV-2), has rapidly spread across the globe and affected every aspect of life of individuals^1^. As of August 30, 2022, almost 601 million cases of COVID-19 have been confirmed worldwide, with over 6 million deaths^2^. During the pandemic, people were requested to reduce social contact and increase telework^3^. This unprecedented situation could be emotionally challenging and it can lead to an increase in suicidality^4,5^.

The impact of the pandemic on suicide is still unclear, and differences based on the inherent cultural and socioeconomic characteristics of countries and individuals may be observed. Previous studies have investigated the relationship between COVID-19 and suicide. While some epidemiological studies have shown an increase in suicides during the COVID-19 period^6,7^, others have shown a decrease or no change in suicides during the same period^8–10^. In addition, one study revealed the fluctuating risk of suicide during the pandemic, showing that national suicide rates declined during the first five months of the pandemic and then increased during the second wave^3^. There are conflicting explanations that partly address these mixed results. Together with the anxiety caused by the fear of infection, social distancing can lead to disconnections between people and the resultant social isolation can be related to increased suicidal behavior^11^. Further, the economic recession along with increased unemployment rates has been consistently suggested as a major risk factor for suicide related to the pandemic^12^. However, suicides have been traditionally recognized to initially decrease during natural disasters—such as epidemics and terrors—owing to the strong social cohesion within communities^13^. Suicides were found to have diminished at the initial stage of influenza, severe acute respiratory syndrome, and Ebola epidemics, although the risk of suicide increased afterward^14^. Moreover, with the increase in the amount of time spent staying at home during the current pandemic, emotional and physical support from family could be beneficial to the mental health of individuals^15^.

There have been current and growing concerns about whether the suicide rate in South Korea (hereafter, Korea) has changed since the COVID-19 outbreak, as the country ranked first in terms of the suicide rate among Organization for Economic Co-operation and Development (OECD) member countries in 2019, with 24.6 suicides per 100,000 persons^16^. In addition, Korea is one of the representative countries that has responded well to the COVID-19 pandemic at the early stage by developing clear guidelines for the public and performing extensive epidemiological investigations, including aggressive diagnostic testing^17^. Thus, investigating the changes in the pattern of suicide rates in Korea during the COVID-19 pandemic will provide significant implications for other countries. In this regard, recently, one epidemiological study investigated suicide numbers during the first 9–15 months of the COVID-19 pandemic in 33 countries stratified by sex and age, including the results of decreased suicide in Korea^18^. However, although psychiatric effects of pandemics on suicide could change during the long-lasting pandemic^3^ and the effects likely differ depending on socioeconomic factors that are closely associated with social inequalities^19^, the studies that have addressed these topics are relatively rare.

Therefore, this study aimed to (1) investigate the national excess suicide attributable to the COVID-19 pandemic in Korea using officially-distributed mortality data, (2) examine the temporal changes in the risk of suicide during the pandemic, and (3) identify the individual-level characteristics associated with (1) and (2), by using a novel and standardized two-stage interrupted time-series model.

## Methods

### Data acquisition

We collected data on daily time-series suicide mortality, weather variables, and confirmed cases of COVID-19 for all 16 regions (shi/do) of Korea, which include seven metropolitan/special cities (shi) and nine provinces (do) within the period 2017–2020. Data on mortality were collected from the Korean National Statistics Office, and suicide was defined as intentional self-poisoning and self-harm based on the International Statistical Classification of Diseases and Related Health Problems 10th Revision (ICD-10) codes X60–X84^20^. The database included information on sex, age, education level, and marital status of the dead; we re-processed raw data to the number of daily suicide deaths stratified by these factors. We obtained the daily mean temperature (℃) and relative humidity (%) data measured by the automated synoptic observing system from the Korean Meteorological Office to consider the potential confounding effects of them on suicide since several studies reported that high temperature and humidity are associated with suicide pattern^21,22^. The COVID-19 confirmed cases data was collected from the Korea Centers for Disease Control and Prevention. Additionally, as our study is a cross-sectional study based on the observational data, we provided the STROBE checklist in Supplementary materials.

### Definition of the period

Although the first imported case of COVID-19 was confirmed in Korea on January 20, 2020, we quantified excess suicide from February 18, 2020 instead of January 20, 2020, because the first wave started when the 31^st^ confirmed case occurred on February 18, 2020 within a religious group called Shincheonji in Daegu^17^. In addition, to examine the heterogeneity among different time periods, we defined two specific periods—waves and plateaus—based on the transmission patterns over time using the following definition; except for the start day (February 18, 2020), waves were defined as the periods wherein the seven-day moving-average of confirmed cases is 100 or more and the rest of the periods were defined as plateaus—the first wave (February 18–March 17, 2020), the first plateau (March 18–August 14, 2020), the second wave (August 15–September 22, 2020), the second plateau (September 23–October 25, 2020) and the third wave (October 26–December 31, 2020). We used a moving-average for the definition of the periods to capture the average change in suicide over time.

### Statistical analyses

We performed a two-stage interrupted time-series analysis to quantify the time-varying excess number and risk for suicide during the COVID-19 pandemic compared with the pre-outbreak period. This is a standardized and cutting-edge approach that has been widely used in previous studies^23,24^.

In the first stage, we conducted an interrupted time-series analysis for 16 regions. An overdispersion test (Table S1) provided evidence of the existence of overdispersion in several regions; therefore, we applied a quasi-Poisson distribution to adjust for it. To estimate the time-varying excess suicide attributable to the pandemic, we included the days from the first COVID-19 case in the model using a constrained quadratic B-spline with four equally spaced knots for the outbreak period. This spline function constrains the excess risk to start from null at the beginning of the outbreak period and then allows it to vary flexibly until the end of the study period. We controlled long-term trends using a linear term for time, seasonality using a cyclic cubic B-spline with four equally spaced knots for the day of the year, and weekly variations in suicide using dummy indicators for the day of the week. To control for the potential confounding effects, we included a term for the daily mean temperature and relative humidity. The daily mean temperature was adjusted using a distributed lag non-linear model (DLNM), which can concurrently consider the non-linear exposure–response and the non-linear lag-response associations^25^. We used a quadratic B-spline for the exposure–response association with three internal knots placed at the 25th, 50th, and 75th percentiles of region-specific temperature distribution^21^. For the lag-response association, we used dummy parameterization-defining intervals with lag 0 and lag 1–2^26^. These modeling specifications were based on previous studies on suicide and temperature^21,26^. The current day (lag0) relative humidity was controlled using a natural cubic B-spline with two degrees of freedom.

In the second stage, we pooled the region-specific estimates and calculated the best linear unbiased predictions (BLUPs) to compute the nationwide excess suicide mortality during the COVID-19 pandemic using a mixed-effects multivariate meta-analysis model. All statistical analyses were performed using the packages *dlnm* and *mixmeta* in R software (version 4.1.0.)^26–28^. For the reproductivity of our study, we provided the R code in Supplementary materials.

### Quantification of excess suicide

Excess death is a term used in epidemiology and public health that refers to the number of deaths during a crisis above and beyond what we would have expected to see under “normal” conditions^29^. First, we calculated the daily region-specific and nationwide excess risks (relative risk, RR) that indicate the excess for each day of the pandemic period with the region-specific BLUPs and pooled estimates from the second-stage meta-analysis model. Then, the daily number of excess suicide was computed as (RR-1)/RR*n, in which *n* is the daily number of suicides, and was aggregated in total for the period February 18–December 31, 2020 in each region and the whole of Korea. We calculated empirical confidence intervals (eCIs) at 95% using 1000 Monte Carlo simulations, assuming a multivariate normal distribution using the point estimates and covariance matrix.

### Sub-group analysis

We repeated all analyses according to individual characteristics: sex, age group (0–39 years, 40–64 years, and 65+ years), education level (< high school, high school, and > high school), and marital status [single, married, and others (divorced or widowed)].

### Sensitivity analyses

To evaluate the robustness of our findings, we performed several sensitivity analyses. First, we repeated the aforementioned analyses except for people aged under 10 years who do not generally have suicidal ideation. Second, we altered the number of internal knots for the cyclic cubic B-spline of the day of the year from four to five. Third, we changed the number and position of internal knots for the exposure–response curve for the DLNM of the mean temperature from three (at the 25th, 50th, and 75th percentiles of temperature) to two (at the 50th and 90th percentiles) and three (at the 10th, 75th, and 90th percentiles). Fourth, the lag period was extended to five, seven, and ten days for the lag-response curve for the DLNM. Finally, we altered the number of internal knots for the natural cubic B-spline of the relative humidity from two to three. These modeling specifications were based on earlier studies^21,24^.

## Results

**Figure 1:**
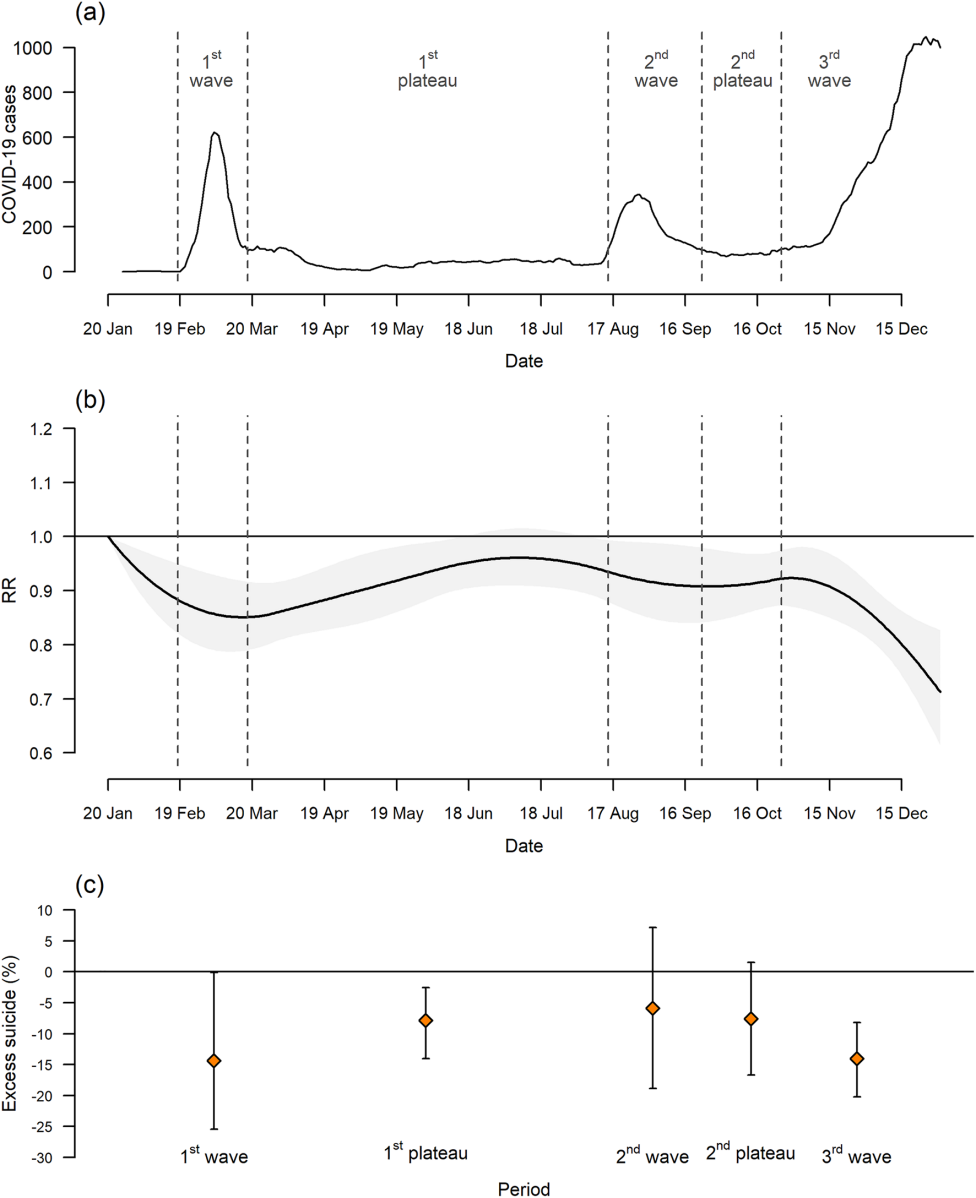
Temporal trend in 7-day moving average of confirmed COVID-19 cases (**a**), excess risk (relative risk, RR) of suicide with a band corresponding to the 95% eCI (**b**), and percent excess of suicide by period (**c**) during the period January 20–December 31, 2020 in Korea. *eCI* empirical confidence interval.

Across the study period (January 1, 2017– December 31, 2020), a total of 53 127 suicide deaths were recorded (4.5% of total deaths) in Korea (Table 1). The 7-day moving average of confirmed COVID-19 cases is displayed in Fig. 1 and the geographical patterns of confirmed cases, average temperature, and average relative humidity are illustrated in Supplementary Fig. S1.

**Table 1:** Characteristics of the 16 regions in Korea within the study period (January 1, 2017–December 31, 2020).

Region (shi/do)^a^	Number of districts (shi/gun/gu)^a^	Population density (/km^2^)	Deaths per 100,000 people	Confirmed COVID-19 cases per 100,000 people^b^	Suicide (%)^c^
Seoul	25	16,375.8	461.4	192	8511 (4.8)
Busan	16	4465.4	665.2	54	3800 (4.3)
Daegu	8	2768.7	573.5	319	2596 (4.7)
Incheon	10	2826.1	516.9	94	3047 (5.0)
Gwangju	5	2936.1	530.1	73	1374 (4.5)
Daejeon	5	2743.9	449.1	57	1577 (5.3)
Ulsan	5	1086.4	406.0	58	1220 (5.9)
Gyunggi	41	1354.3	463.6	105	12,448 (5.2)
Gangwon	18	92.7	777.1	77	1994 (4.2)
Chungbuk	11	221.1	715.0	71	1866 (4.1)
Chungnam	15	292.3	745.3	71	3148 (4.7)
Jeonbuk	14	227.3	795.9	46	2116 (3.6)
Jeonnam	22	152.6	913.6	30	1998 (2.9)
Gyungbuk	23	141.4	828.3	90	3021 (3.4)
Gyungnam	18	323.3	667.1	39	3588 (4.0)
Jeju	2	377.0	591.3	60	783 (5.0)
Korea	229	527.6	633.7	110	53,127 (4.5)

^a^The 16 regions (shi/do) is the first-level administrative region of Korea and the 229 districts (shi/gun/gu) is the second-level administrative region in Korea.

^b^Confirmed COVID-19 cases per 100,000 people indicates the cumulative COVID-19 confirmed cases during the period January 20–December 31, 2020.

^c^Proportion of suicide was calculated by dividing the number of suicide deaths by the number of total deaths for the study period.

During the period February 18–December 31, 2020, the estimated decrease in suicidal cases was 1 192 [95% empirical confidence interval (eCI): 515, 1 968] compared to the expected baseline number and it accounted for 9.5% (95% eCI: 3.8%, 15.6%) decrease in suicide during the same period (Table 2). Figure 1b,c demonstrate temporal variations in excess risk (relative risk, RR) of suicide and % excess of suicide attributable to the pandemic. The excess risk of suicide has declined from the initial pandemic, and this decreasing pattern was pronounced during the first and third waves: the corresponding % decrease in excess death were 14.4% (95% eCI: 0.1%, 25.4%) and 14.0% (95% eCI: 8.2%, 20.2%), individually. The corresponding period-specific number of excess suicides is provided in Table S2.

**Table 2:** Total number, estimated excess (95% eCI), and percent excess (95% eCI) of suicide during the period February 18–December 31, 2020 in Korea by region.

	Total	Excess	Percent excess (%)
Seoul	1902	−92 (−227, 28)	−4.6 (−10.7, 1.5)
Busan	803	−100 (−169, −44)	−11.1 (−17.4, −5.2)
Daegu	551	−63 (−114, −22)	−10.3 (−17.1, −3.9)
Incheon	664	−73 (−131, −25)	−9.9 (−16.5, −3.7)
Gwangju	286	−36 (−63, −13)	−11.1 (−18.1, −4.3)
Daejeon	340	−39 (−71, −11)	−10.3 (−17.3, −3.3)
Ulsan	266	−31 (−57, −10)	−10.4 (−17.6, −3.5)
Gyunggi	2730	−331 (−515, −168)	−10.8 (−15.9, −5.8)
Gangwon	445	−37 (−78, −4)	−7.6 (−14.9, −0.9)
Chungbuk	376	−46 (−82, −15)	−10.8 (−18.0, −3.9)
Chungnam	681	−93 (−155, −43)	−12.0 (−18.6, −6.0)
Jeonbuk	455	−44 (−86, −10)	−8.8 (−15.9, −2.2)
Jeonnam	452	−27 (−68, 6)	−5.7 (−13.0, 1.3)
Gyungbuk	659	−70 (−128, −23)	−9.6 (−16.3, −3.3)
Gyungnam	729	−101 (−166, −47)	−12.1 (−18.6, −6.1)
Jeju	174	−23 (−40, −9)	−11.5 (−18.8, −4.8)
Korea	11,513	−1204 (−2120, −456)	−9.5 (−15.6, −3.8)

We computed empirical confidence intervals (eCIs) at 95% by Monte Carlo simulation.

*eCI* empirical confidence interval.

**Figure 2:**
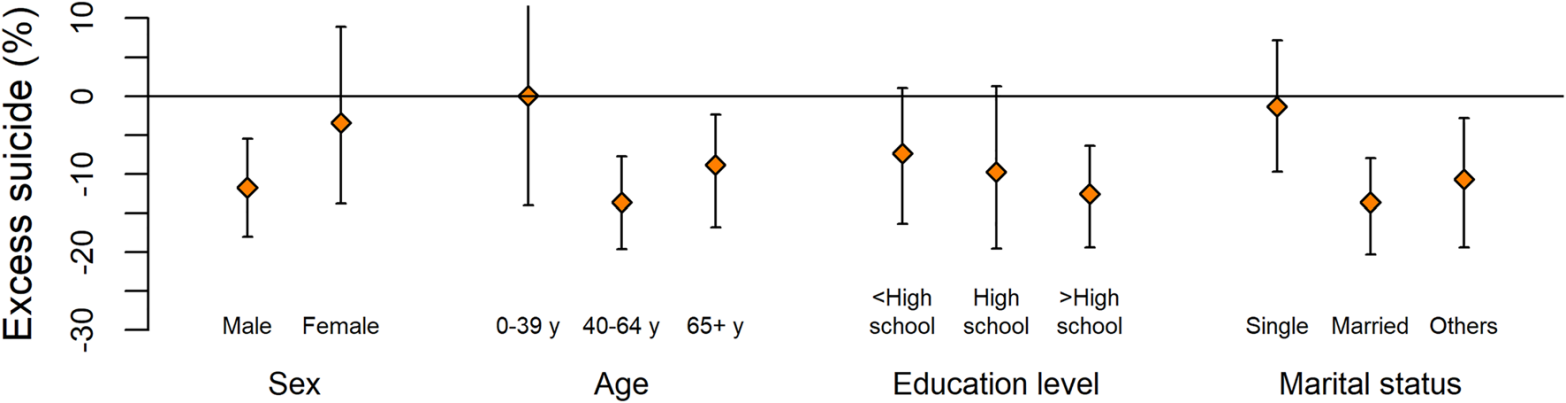
Percent excess and 95% CI in suicide during the period February 18–December 31, 2020 in Korea by sex, age, education level, and marital status. In terms of marital status, others include divorced and widowed people. *CI* confidence interval.

**Figure 3:**
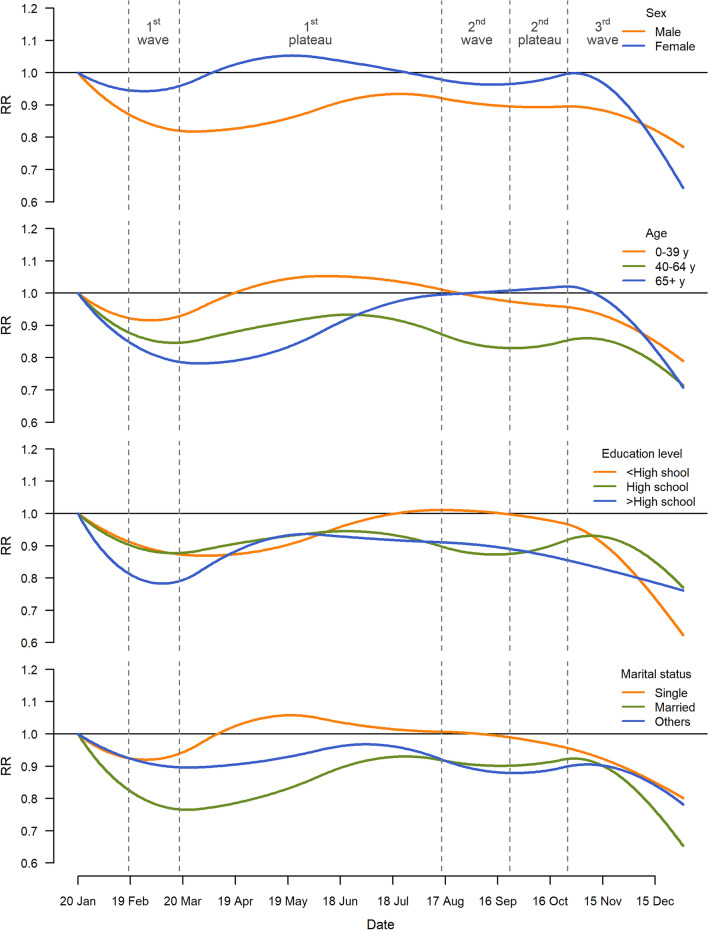
Temporal trend in excess risk (relative risk, RR) of suicide in Korea by sex, age, education level, and marital status during the period January 20–December 31, 2020. In terms of marital status, others include divorced and widowed people.

We investigated the heterogeneous impacts of the pandemic on suicide across sex, age groups, education level, and marital status. During the period February 18–December 31, 2020, the decrease in suicide mortality was more evident in individuals who were male, middle-aged (aged 40–64 years), highly educated (more than high school), and married, with the % of excess suicide, based on the central estimates: −11.7% (95% eCI: −18.0%, −5.5%), −13.7% (95% eCI: −19.6%, −7.8%), −12.6% (95% eCI: −19.4%, −6.4%), and −13.6% (95% eCI: −20.3%, −8.0%) individually (Fig. 2). The corresponding number of excess suicides to Fig. 2 are provided in the Supplementary Tables S3–S6. Figure 3 demonstrates the temporal variations in the RR of suicide during the pandemic by individual characteristics. It indicates that the decrease in suicide risk was generally more evident in individuals who were male, middle-aged, highly educated, or married than in the general population across the entire pandemic period.

We applied different study populations and modeling specifications to evaluate the robustness of our main results. The sensitivity analyses showed that our main results are robust, showing a decrease in suicide during the period February 18–December 31, 2020 (Supplementary Fig.S2 and Supplementary Table S7–S11).

## Discussion

This study investigated the changes in suicide deaths during the COVID-19 pandemic in Korea using nationwide mortality data. We revealed that suicide death decreased by 9.5% (95% eCI: 3.8%, 15.6%) during the pandemic compared to the expected baseline number from the pre-pandemic period. Further, the decrease in suicide was prominent during the first and third wave of the COVID-19 outbreak and in male, middle-aged, highly educated, or married people.

We found that suicide risk decreased during the COVID-19 pandemic period. The results generally align with those of previous studies, in which either a decrease or no significant change in suicide during the pandemic was observed. A study conducted in 33 countries suggested the evidence of lower-than-expected number of suicides in general. One cohort study in Connecticut, in the United States, showed that the age-adjusted suicide rate declined by 13% during the lockdown period compared to the 5-year average^8^. A study in Japan reported that suicide rates declined by 14% during the first five months of the pandemic (February to June, 2020), and then increased by 16% during the second wave (July to October, 2020) with a larger increase among females (37%) and children and adolescents (49%)^3^.

The “honeymoon effect” can be a plausible explanation for the decrease in the suicide during the initial stage of the pandemic. The honeymoon effect indicates the social phenomenon that during external threats—such as wars or epidemics—social cohesion increases temporarily and it may mitigate the risk of suicide^13,14^. We conjecture that people generated strong community consciousness and social cohesion to overcome the current public health crisis^30^, and this is associated with an overall decrease in suicide during the pandemic period. We also found lower suicide risk during the infection waves and this result can be also explained by the aforementioned perspective; i.e., a higher level of social distancing and the anxiety attributable to the rapidly increasing number of confirmed cases during the waves may have further increased social cohesion and altruism, resulting in decreased suicidal behavior. However, according to previous studies including the Japanese study we mentioned earlier^3,14^, the increase in social connectedness may not be permanent, and suicide rates can increase after the pandemic or at a later stage of the pandemic. Therefore, the excess suicide attributable to the pandemic should be consistently examined even after the pandemic, and public health specialists need to be prepared to deal with the possible long-term detrimental effects of the pandemic on suicide.

In the Korean context, additional explanations could have been attributed to the decline in suicide during the pandemic period. Korea is one of the countries with world-leading information and communication technology (ICT) infrastructure^31^. Therefore, the Korean society could implement the transition to telework and distance education quickly at the initial stage of the pandemic. Along with a strict social distancing measures, the prevalent introduction of telework and distance education made people stay at home for a longer time and the resultant increase in family support may have been a major factor that reduced social isolation and suicide risk. Moreover, we also surmise that nationwide financial support, called the COVID-19 Economic Impact Payment (EIP), offered by the Korean government during the pandemic could be associated with reduced financial distress, which is a major risk factor of suicide in Korea^32^. Unlike other countries, in particular, the Korean government adopted an electronic payment technique involving vouchers and cash transfers in local currencies and credit cards to supply the fund. In addition, the available period and location of the usage were limited in order to increase the effectiveness of the EIP. As a result, consumption expenditure increased by 10.4% after May^33^, and lower-income households experienced larger increases in expenditure^34^.

We confirmed that the effects of the pandemic on suicide were disproportionately distributed by individual characteristics that are closely associated with socioeconomic inequalities. We postulate that different patterns of social relationships between males and females can partly explain the difference in the effect of the pandemic on suicide. Compared to females who have bigger social networks and receive social support from various sources^35^, males generally derive lower psychobiological benefits from social capital and are more susceptible to social isolation^36^. Thus, increased family support and reduced social isolation due to the increase in telework and social distancing can result in a decrease in suicide risk among males during the pandemic. Moreover, we found a larger decrease in suicide among middle-aged populations during the pandemic than among other age groups. In Korea, the middle-aged population is generally the most active in socioeconomic activities among all age groups^37^ and severe stress from the workplace and social relationships is a dominant risk factor for suicide^38^. We infer that the increased telework and the resultant decrease in stress from socioeconomic activities were beneficial in reducing the suicide risk among the middle-aged populations during the pandemic.

Our study also found a more evident decrease in suicide risk among people who were highly educated and married compared to the general population during the pandemic. We postulate that the disproportionate decreases in suicide attributable to the pandemic by education level are associated with disparate accessibility to accurate information on the COVID-19 pandemic. Highly educated people are more likely to be exposed to accurate information regarding the pandemic and are aware of suitable countermeasures through social media and the literature^39^, which could alleviate the unnecessary anxiety and stress that can bring about suicidal ideation. In terms of the marital status, as opposed to single or non-married populations, married people are more likely to receive emotional support from their family during the pandemic, which can reduce the suicide risk. In addition, as the age of first marriage continues to increase in Korea due to socioeconomic factors, there is a social phenomenon wherein economically stable people are more likely to get married^40^. Further, people with high education have a larger probability to have more stable work conditions and higher incomes. Therefore, there is a possibility that highly educated and married people were less susceptible to the COVID-19 pandemic, and related economic recession and negative changes in livelihood, such as unemployment^41^; however, these explanations should be examined in-depth by future studies.

We acknowledge several limitations in this study. First, because the mortality dataset was publicly available only until 2020, we were unable to consider the extended study period (i.e. 2021 and 2022). Since the pandemic has been prolonging for more than two years and the trend in suicide could change, further studies should be conducted to examine long-term effects of the pandemic on suicide. Second, our study is based on suicide mortality data, not the suicidal behavior data; thus, we were unable to consider the changes in various suicidal events (e.g. suicidal ideation and suicidal attempts), and it can underestimate the overall effects of the pandemic on suicide-related events. Despite these limitations, a clear strength of this study is the application of a state-of-the-art method i.e. a two-stage interrupted time-series design that allows a flexible estimation of the excess mortality in Korea, after adjusting for temporal trends and variations in temperature and humidity. In addition, our results regarding Korea can provide implications to global public health policymakers implying that proper responses to the pandemic can be effective in reducing potential problems in mental health. Finally, by revealing the different effects of the pandemic on suicide by individual characteristics, we can identify potential risk factors which are related to socioeconomic disparities and provide evidence for the differentiated interventions in order to reduce the detrimental effects of the pandemic on suicide.

## Conclusion

To the best of our knowledge, this is the first nationwide study to examine the time-varying excess suicide death attributable to the COVID-19 pandemic in Korea and its socio-demographic variations. Our results can provide timely and scientific evidence for establishing public health interventions and also suggest the prioritization of resource allocation to cater to the mental health of individuals according to the different vulnerabilities by individual-level characteristics.

## Supplementary Information

Supplementary Information.

## Data Availability

The dataset on mortality is publicly available in the Korean National Statistics Office, https://kostat.go.kr. The datasets on meteorological variables (mean temperature and relative humidity) are publicly available in the Korean Meteorological Office, https://www.weather.go.kr. The dataset on COVID-19 confirmed cases is publicly available in the Korea Centers for Disease Control and Prevention, https://www.kdca.go.kr.
