# Supplementary material for: Excess suicide attributable to the COVID-19 pandemic and social disparities in South Korea
Source: Sci Rep. 2022 Nov 1;12:18390. doi: 10.1038/s41598-022-22751-7 (PMC9626586; doi:10.1038/s41598-022-22751-7)
Supplement: Supplementary file 1 — Supplementary Information. [file 41598_2022_22751_MOESM1_ESM.docx]

**Supplementary materials**

**Title.** Excess suicide attributable to the COVID-19 pandemic and social disparities in South Korea

**Authors.** Jieun Min^1^, Jieun Oh^1^, Soo In Kim^2^, Cinoo Kang^1^, Eunhee Ha^3,4^, Ho Kim^1,5^, and Whanhee Lee^4,6*^

**Affiliations**

^1^Department of Public Health Science, Graduate School of Public Health, Seoul National University, Seoul, Republic of Korea.

^2^Department of Psychiatry, Ewha Womans University Mokdong Hospital, Ewha Womans University College of Medicine, Seoul, Republic of Korea.

^3^Department of Occupational and Environmental Medicine, Ewha Womans University College of Medicine, Seoul, Republic of Korea.

^4^Institute of Ewha-SCL for Environmental Health (IESEH), Ewha Womans University College of Medicine, Seoul, Republic of Korea.

^5^Institute of Sustainable Development, Seoul National University, Seoul, Republic of Korea. ^6^School of Biomedical Convergence Engineering, College of Information and Biomedical Engineering, Pusan National University, Yangsan, Republic of Korea

^*^**Correspondence:** Whanhee Lee, School of Biomedical Convergence Engineering, College of Information and Biomedical Engineering, Pusan National University, Yangsan, Republic of Korea 50612, E-mail: [whanhee.lee@pusan.ac.kr](mailto:whanhee.lee@pusan.ac.kr)

**Contents**

1. Table S1–11

2. Fig. S1–2

3. STROBE Statement

4. R code for statistical analyses

**1. Table S1–11**

**Table S1. Results of the overdispersion test by region**

|  | **Z** | **p-value** |
| --- | --- | --- |
| Seoul | **4.10** | **<0.001** |
| Busan | **0.63** | **0.264** |
| Daegu | **1.93** | **0.027** |
| Incheon | **1.56** | **0.059** |
| Gwangju | **1.35** | **0.889** |
| Daejeon | **1.65** | **0.450** |
| Ulsan | **0.06** | **0.476** |
| Gyunggi | **6.48** | **<0.001** |
| Gangwon | **0.84** | **0.202** |
| Chungbuk | **0.45** | **0.326** |
| Chungnam | **1.52** | **0.064** |
| Jeonbuk | **0.36** | **0.358** |
| Jeonnam | **-0.20** | **0.581** |
| Gyungbuk | **0.24** | **0.404** |
| Gyungnam | **1.33** | **0.092** |
| Jeju | **1.86** | **0.031** |

**Table S2.** Number of suicide and estimated excess (with 95% eCI^a^) during the period February 18–December 31, 2020 by region and period

|  | 1^st^ wave | |  | 1^st^ plateau | |  | 2^nd^ wave | |  | 2^nd^ plateau | |  | 3^rd^ wave | |
| --- | --- | --- | --- | --- | --- | --- | --- | --- | --- | --- | --- | --- | --- | --- |
|  | Total | Excess |  | Total | Excess |  | Total | Excess |  | Total | Excess |  | Total | Excess |
| Seoul | 168 | -23 (-49, 2) |  | 922 | -32 (-104, 27) |  | 248 | 6 (-27, 31) |  | 188 | 0 (-18, 14) |  | 376 | -42 (-74, -14) |
| Busan | 73 | -12 (-26, 1) |  | 413 | -37 (-72, -8) |  | 86 | -15 (-32, -2) |  | 84 | -12 (-24, -3) |  | 147 | -24 (-39, -13) |
| Daegu | 51 | -8 (-18, 1) |  | 275 | -28 (-54, -8) |  | 61 | -3 (-15, 5) |  | 67 | -6 (-15, 2) |  | 97 | -18 (-28, -9) |
| Incheon | 80 | 0 (-13, 12) |  | 321 | -32 (-62, -9) |  | 72 | -9 (-23, 2) |  | 65 | -9 (-18, -1) |  | 126 | -23 (-36, -13) |
| Gwangju | 25 | -5 (-10, 0) |  | 141 | -12 (-26, -1) |  | 23 | -4 (-10, 0) |  | 30 | -4 (-9, -1) |  | 67 | -10 (-17, -5) |
| Daejeon | 29 | -4 (-9, 1) |  | 161 | -17 (-31, -5) |  | 42 | -2 (-9, 5) |  | 32 | -2 (-7, 1) |  | 76 | -14 (-23, -7) |
| Ulsan | 33 | -5 (-12, 2) |  | 136 | -11 (-24, 0) |  | 27 | -4 (-10, 1) |  | 20 | -2 (-6, 0) |  | 50 | -9 (-15, -5) |
| Gyunggi | 240 | -55 (-93, -19) |  | 1,321 | -148 (-250, -67) |  | 353 | -11 (-56, 25) |  | 279 | -20 (-48, 4) |  | 537 | -98 (-146, -59) |
| Gangwon | 36 | -5 (-13, 2) |  | 227 | -15 (-36, 1) |  | 58 | -2 (-13, 7) |  | 47 | -3 (-9, 2) |  | 77 | -12 (-20, -5) |
| Chungbuk | 41 | -5 (-13, 2) |  | 169 | -18 (-35, -4) |  | 50 | -5 (-15, 3) |  | 48 | -5 (-12, 0) |  | 68 | -12 (-19, -6) |
| Chungnam | 54 | -12 (-23, -2) |  | 342 | -34 (-66, -9) |  | 79 | -12 (-28, 0) |  | 67 | -9 (-18, -2) |  | 139 | -25 (-39, -14) |
| Jeonbuk | 39 | -8 (-17, 0) |  | 237 | -15 (-37, 2) |  | 63 | -5 (-18, 4) |  | 43 | -4 (-10, 1) |  | 73 | -11 (-18, -5) |
| Jeonnam | 45 | -4 (-13, 4) |  | 211 | -14 (-34, 3) |  | 67 | 2 (-10, 11) |  | 54 | -1 (-8, 5) |  | 75 | -10 (-18, -4) |
| Gyungbuk | 50 | -9 (-19, 0) |  | 345 | -26 (-58, -1) |  | 83 | -10 (-26, 3) |  | 65 | -7 (-16, 0) |  | 116 | -18 (-30, -9) |
| Gyungnam | 56 | -14 (-26, -4) |  | 375 | -40 (-74, -12) |  | 84 | -10 (-26, 3) |  | 69 | -9 (-18, -1) |  | 145 | -28 (-43, -16) |
| Jeju | 23 | -5 (-11, 0) |  | 82 | -7 (-15, 0) |  | 20 | -3 (-8, 1) |  | 13 | -2 (-4, 0) |  | 36 | -6 (-10, -3) |
| Korea | 1,043 | -175 (-356, -1) |  | 5,678 | -486 (-925, -147) |  | 1,416 | -89 (-329, 95) |  | 1,171 | -96 (-235, 17) |  | 2,205 | -359 (-558, -197) |

eCI, empirical confidence interval ^a^We computed empirical confidence intervals (eCIs) at 95% by Monte Carlo simulation.

**Table S3.** Number of suicide and estimated excess (with 95% eCI^a^)
during the period February 18–December 31, 2020 by region and sex

|  | Males | |  | Females | |
| --- | --- | --- | --- | --- | --- |
|  | Total | Excess |  | Total | Excess |
| Seoul | 1,241 | -81 (-177, 7) |  | 661 | -4 (-78, 66) |
| Busan | 558 | -80 (-133, -29) |  | 245 | -4 (-36, 25) |
| Daegu | 368 | -63 (-104, -28) |  | 183 | -9 (-34, 14) |
| Incheon | 455 | -99 (-149, -56) |  | 209 | 1 (-26, 26) |
| Gwangju | 197 | -22 (-45, -2) |  | 89 | -1 (-16, 12) |
| Daejeon | 229 | -47 (-74, -22) |  | 111 | -5 (-21, 10) |
| Ulsan | 190 | -22 (-45, -1) |  | 76 | 1 (-10, 11) |
| Gyunggi | 1,880 | -243 (-385, -109) |  | 850 | -91 (-188, 4) |
| Gangwon | 303 | -37 (-70, -9) |  | 142 | 2 (-17, 19) |
| Chungbuk | 274 | -54 (-84, -27) |  | 102 | 3 (-12, 15) |
| Chungnam | 459 | -79 (-129, -34) |  | 222 | -4 (-32, 23) |
| Jeonbuk | 338 | -29 (-63, 2) |  | 117 | -4 (-19, 10) |
| Jeonnam | 333 | -48 (-84, -17) |  | 119 | 2 (-15, 17) |
| Gyungbuk | 433 | -61 (-109, -18) |  | 226 | 6 (-21, 32) |
| Gyungnam | 529 | -70 (-122, -22) |  | 200 | -20 (-48, 6) |
| Jeju | 125 | -18 (-34, -5) |  | 49 | -2 (-9, 5) |
| Korea | 7,912 | -1,053 (-1,742, -459) |  | 3.601 | -128 (-577, 295) |

eCI, empirical confidence interval ^a^We computed empirical confidence intervals (eCIs) at 95% by Monte Carlo simulation.

**Table S4.** Number of suicide and estimated excess (with 95% eCI^a^) during the period February 18–December 31, 2020
by region and age group

|  | 0–39 y | |  | 40–64 y | |  | 65+ y | |
| --- | --- | --- | --- | --- | --- | --- | --- | --- |
|  | Total | Excess |  | Total | Excess |  | Total | Excess |
| Seoul | 638 | 54.7 (-26, 126) |  | 799 | -111 (-174, -56) |  | 465 | -45 (-91, -9) |
| Busan | 182 | -4 (-37, 24) |  | 395 | -44 (-76, -15) |  | 226 | -27 (-52, -8) |
| Daegu | 168 | 7 (-23, 32) |  | 248 | -46 (-71, -26) |  | 135 | -9 (-27, 4) |
| Incheon | 210 | 22 (-11, 51) |  | 330 | -49 (-80, -23) |  | 124 | -24 (-41, -12) |
| Gwangju | 89 | -8 (-28, 8) |  | 134 | -14 (-29, -2) |  | 63 | -5 (-16, 4) |
| Daejeon | 115 | 0 (-22, 19) |  | 140 | -27 (-41, -16) |  | 85 | -12 (-26, -3) |
| Ulsan | 74 | 4 (-11, 16) |  | 150 | -19 (-34, -6) |  | 42 | -7 (-12, -4) |
| Gyunggi | 828 | 2 (-104, 98) |  | 1,267 | -258 (-355, -169) |  | 635 | -66 (-126, -16) |
| Gangwon | 85 | -2 (-20, 14) |  | 213 | -26 (-46, -9) |  | 147 | -8 (-26, 4) |
| Chungbuk | 97 | -2 (-23, 14) |  | 164 | -30 (-46, -17) |  | 115 | 3 (-12, 12) |
| Chungnam | 163 | -27 (-61, 2) |  | 315 | -53 (-83, -27) |  | 203 | -12 (-35, 5) |
| Jeonbuk | 108 | -2 (-22, 16) |  | 204 | -22 (-41, -6) |  | 143 | -14 (-32, -2) |
| Jeonnam | 92 | -2 (-20, 14) |  | 202 | -32 (-52, -16) |  | 158 | -9 (-29, 4) |
| Gyungbuk | 139 | -8 (-35, 15) |  | 313 | -44 (-72, -20) |  | 207 | -26 (-52, -9) |
| Gyungnam | 182 | -32 (-69, 1) |  | 362 | -56 (-89, -27) |  | 185 | -28 (-52, -12) |
| Jeju | 42 | -4 (-14, 5) |  | 88 | -12 (-22, -4) |  | 44 | 2 (-6, 7) |
| Korea | 3,212 | 1 (-523, 450) |  | 5,324 | -843 (-1,302, -449) |  | 2,977 | -288 (-601, -73) |

eCI, empirical confidence interval ^a^We computed empirical confidence intervals (eCIs) at 95% by Monte Carlo simulation.

**Table S5.** Number of suicide and estimated excess (with 95% eCI^a^) during the period February 18–December 31, 2020
by region and education level

|  | <High school | |  | High school | |  | >High school | |
| --- | --- | --- | --- | --- | --- | --- | --- | --- |
|  | Total | Excess |  | Total | Excess |  | Total | Excess |
| Seoul | 452 | -11 (-68, 35) |  | 638 | -4 (-70, 58) |  | 613 | -84 (-145, -34) |
| Busan | 235 | -11 (-46, 14) |  | 322 | -59 (-106, -17) |  | 202 | -38 (-60, -21) |
| Daegu | 149 | -18 (-41, 1) |  | 196 | -11 (-40, 14) |  | 178 | -25 (-43, -10) |
| Incheon | 175 | -20 (-47, 2) |  | 263 | 1 (-33, 30) |  | 146 | -25 (-40, -12) |
| Gwangju | 77 | -6 (-18, 4) |  | 92 | -12 (-28, 2) |  | 106 | -15 (-26, -6) |
| Daejeon | 93 | -10 (-25, 2) |  | 126 | -5 (-26, 12) |  | 98 | -13 (-25, -3) |
| Ulsan | 56 | -5 (-15, 2) |  | 112 | -14 (-34, 3) |  | 87 | -14 (-22, -6) |
| Gyunggi | 675 | -78 (-167, -6) |  | 1,010 | -164 (-275, -58) |  | 811 | -111 (-188, -46) |
| Gangwon | 166 | -11 (-37, 8) |  | 152 | -10 (-34, 10) |  | 98 | -13 (-24, -4) |
| Chungbuk | 129 | -14 (-33, 2) |  | 142 | -16 (-41, 6) |  | 92 | -15 (-25, -7) |
| Chungnam | 245 | -27 (-65, 3) |  | 230 | -36 (-72, -6) |  | 168 | -17 (-35, -2) |
| Jeonbuk | 156 | -10 (-34, 10) |  | 146 | -15 (-38, 5) |  | 117 | -18 (-31, -7) |
| Jeonnam | 179 | -3 (-31, 16) |  | 166 | -3 (-29, 19) |  | 87 | -12 (-22, -4) |
| Gyungbuk | 243 | -14 (-50, 13) |  | 250 | -40 (-78, -7) |  | 148 | -23 (-38, -10) |
| Gyungnam | 227 | -22 (-56, 5) |  | 262 | -52 (-95, -16) |  | 201 | -29 (-51, -11) |
| Jeju | 44 | -3 (-10, 2) |  | 59 | -11 (-22, -2) |  | 51 | -8 (-13, -4) |
| Korea | 3,301 | -263 (-644, 33) |  | 4,166 | -449 (-1,013, 53) |  | 3,203 | -460 (-769, -218) |

eCI, empirical confidence interval ^a^We computed empirical confidence intervals (eCIs) at 95% by Monte Carlo simulation.

**Table S6.** Number of suicide and estimated excess (with 95% CI^a^) during the period February 18–December 31, 2020
by region and marital status

|  | Single | |  | Married | |  | Others^b^ | |
| --- | --- | --- | --- | --- | --- | --- | --- | --- |
|  | Total | Excess |  | Total | Excess |  | Total | Excess |
| Seoul | 783 | 23 (-45, 80) |  | 691 | -85 (-168, -18) |  | 420 | -25 (-81, 21) |
| Busan | 273 | -8 (-36, 15) |  | 324 | -71 (-117, -33) |  | 206 | -10 (-41, 13) |
| Daegu | 187 | -5 (-24, 11) |  | 207 | -40 (-72, -15) |  | 155 | -18 (-46, 1) |
| Incheon | 238 | -5 (-29, 16) |  | 239 | -36 (-70, -8) |  | 186 | -28 (-61, -3) |
| Gwangju | 99 | -1 (-12, 8) |  | 111 | -22 (-40, -8) |  | 76 | 0 (-16, 11) |
| Daejeon | 126 | -1 (-15, 11) |  | 114 | -25 (-45, -9) |  | 99 | -17 (-35, -2) |
| Ulsan | 95 | -2 (-12, 7) |  | 111 | -21 (-40, -7) |  | 60 | -4 (-18, 6) |
| Gyunggi | 952 | -13 (-94, 55) |  | 1,116 | -156 (-263, -53) |  | 661 | -151 (-239, -73) |
| Gangwon | 123 | -6 (-18, 5) |  | 186 | -17 (-44, 5) |  | 136 | -5 (-27, 10) |
| Chungbuk | 110 | -3 (-15, 8) |  | 152 | -30 (-54, -12) |  | 114 | -18 (-40, -1) |
| Chungnam | 200 | -5 (-26, 12) |  | 278 | -63 (-103, -27) |  | 202 | -24 (-57, 1) |
| Jeonbuk | 136 | -4 (-18, 8) |  | 211 | -22 (-54, 3) |  | 108 | -7 (-27, 7) |
| Jeonnam | 133 | -2 (-16, 10) |  | 195 | -13 (-41, 10) |  | 123 | 2 (-19, 17) |
| Gyungbuk | 193 | -7 (-27, 9) |  | 287 | -53 (-98, -18) |  | 177 | -10 (-37, 12) |
| Gyungnam | 228 | -13 (-38, 7) |  | 309 | -62 (-107, -24) |  | 192 | -36 (-71, -9) |
| Jeju | 55 | -2 (-9, 3) |  | 68 | -9 (-20, -1) |  | 51 | -3 (-14, 4) |
| Korea | 3,931 | -53 (-423, 262)-5 |  | 4,599 | -727 (-1,169, -399) |  | 2,966 | -355 (-715, -87) |

eCI, empirical confidence interval ^a^We computed empirical confidence intervals (eCIs) at 95% by Monte Carlo simulation.
^b^Others include divorced and widowed people in terms of marital status.

**Table S7.** Sensitivity analysis with study population aged 10 or more:
Total number, estimated excess (95% eCI^a^), and percent excess
(95% eCI^a^) of suicide during the period February 18–December 31, 2020 by region

|  | Total | Excess | Percent excess (%) |
| --- | --- | --- | --- |
| Seoul | 1,901 | -87 (-217, -24) | -4.4 (-10.2, 1.3) |
| Busan | 802 | -98 (-167, -39) | -10.9 (-17.2, -4.6) |
| Daegu | 551 | -62 (-113, -21) | -10.1 (-17.0, -3.7) |
| Incheon | 664 | -74 (-132, -26) | -10.0 (-16.6, -3.8) |
| Gwangju | 286 | -35 (-62, -13) | -11.0 (-17.8, -4.4) |
| Daejeon | 340 | -38 (-73, -12) | -10.1 (-17.6, -3.5) |
| Ulsan | 266 | -31 (-57, -10) | -10.5 (-17.8, -3.8) |
| Gyunggi | 2,730 | -325 (-510, -171) | -10.6 (-15.7, -5.9) |
| Gangwon | 445 | -36 (-76, -4) | -7.5 (-14.6, -0.9) |
| Chungbuk | 376 | -45 (-81, -15) | -10.6 (-17.8, -3.8) |
| Chungnam | 681 | -91 (-148, -40) | -11.8 (-18.9, -5.5) |
| Jeonbuk | 455 | -43 (-85, -9) | -8.7 (-15.8, -2.0) |
| Jeonnam | 452 | -27 (-68, 6) | -5.6 (-13.1, 1.3) |
| Gyungbuk | 659 | -27 (-68, 6) | -9.7 (-16.4, -3.4) |
| Gyungnam | 729 | -70 (-129, -23) | -12.0 (-18.3, -5.6) |
| Jeju | 174 | -22 (-40, -9) | -11.4 (-18.6, -4.7) |
| Korea | 11,511 | -1,185 (-1,983, -458) | -9.3 (-14.7, -3.8) |

eCI, empirical confidence interval ^a^We computed empirical confidence intervals (eCIs) at 95% by Monte Carlo simulation.

**Table S8.** Sensitivity analysis with five knots in seasonality spline term:
Total number, estimated excess (95% eCI^a^), and percent excess
(95% eCI^a^) of suicide during the period February 18–December 31, 2020 by region

|  | Total | Excess | Percent excess (%) |
| --- | --- | --- | --- |
| Seoul | 1,902 | -96 (-228, 12) | -4.8 (-9.9, 1.2) |
| Busan | 803 | -96 (-163, -43) | -10.7 (-16.0, -4.5) |
| Daegu | 551 | -58 (-106, -20) | -9.6 (-15.3, -3.0) |
| Incheon | 664 | -67 (-124, -22) | -9.2 (-14.9, -2.8) |
| Gwangju | 286 | -33 (-59, -13) | -10.4 (-16.3, -3.5) |
| Daejeon | 340 | -35 (-67, -9) | -9.4 (-15.8, -2.4) |
| Ulsan | 266 | -31 (-56, -11) | -10.4 (-16.6, -3.4) |
| Gyunggi | 2,730 | -331 (-521, -176) | -10.8 (-15.3, -5.6) |
| Gangwon | 445 | -36 (-74, -5) | -7.4 (-13.5, -0.5) |
| Chungbuk | 376 | -42 (-76, -14) | -9.9 (-16.0, -3.1) |
| Chungnam | 681 | -90 (-149, -43) | -11.7 (-17.1, -5.6) |
| Jeonbuk | 455 | -43 (-82, -12) | -8.7 (-14.6, -1.9) |
| Jeonnam | 452 | -33 (-72, -1) | -6.7 (-13.1, 0.3) |
| Gyungbuk | 659 | -70 (-126, -26) | -9.7 (-15.3, -3.4) |
| Gyungnam | 729 | -99 (-162, -49) | -11.9 (-17.4, -5.8) |
| Jeju | 174 | -22 (-38, -8) | -11.4 (-18.0, -4.9) |
| Korea | 11,513 | -1,182 (-2,110, -438) | -9.3 (-14.6, -3.2) |

eCI, empirical confidence interval ^a^We computed empirical confidence intervals (eCIs) at 95% by Monte Carlo simulation.

**Table S9.** Sensitivity analysis with different position of knots of exposure-response association in temperature basis term:
Total number, estimated excess (95% eCI^a^), and percent excess (95% eCI^a^) of suicide during period February 18–December 31, 2020
by region

|  | Total | 50^th^ and 90^th^ percentile | |  | 10^th,^ 75^th^, and 90^th^ percentile | |
| --- | --- | --- | --- | --- | --- | --- |
|  |  | Excess | Percent excess (%) |  | Excess | Percent excess (%) |
| Seoul | 1,902 | -85 (-208, 45) | -4.3 (-10.1, 1.8) |  | -85 (-209, 38) | -4.3 (-10.3, 2.1) |
| Busan | 803 | -99 (-163, -32) | -11.0 (-17.0, -4.4) |  | -98 (-164, -37) | -10.9 (-17.8, -4.2) |
| Daegu | 551 | -60 (-108, -13) | -9.9 (-16.5, -2.8) |  | -60 (-110, -16) | -9.9 (-17.0, -2.7) |
| Incheon | 664 | -74 (-129, -18) | -10.1 (-16.5, -3.1) |  | -76 (-135, -25) | -10.2 (-16.9, -3.7) |
| Gwangju | 286 | -37 (-64, -11) | -11.6 (-18.3, -4.5) |  | -38 (-65, -13) | -11.7 (-18.8, -4.3) |
| Daejeon | 340 | -38 (-70, -6) | -10.0 (-17.2, -2.3) |  | -40 (-73, -10) | -10.5 (-18.1, -2.9) |
| Ulsan | 266 | -32 (-59, -10) | -10.6 (-18.0, -3.2) |  | -32 (-59, -9) | -10.9 (-18.6, -3.2) |
| Gyunggi | 2,730 | -321 (-495, -135) | -10.5 (-15.5, -5.2) |  | -330 (-515, -163) | -10.8 (-15.9, -5.6) |
| Gangwon | 445 | -35 (-72, 4) | -7.2 (-14.3, 0.3) |  | -32 (-72, 6) | -6.8 (-14.1, 0.7) |
| Chungbuk | 376 | -46 (-81, -11) | -10.9 (-17.9, -3.4) |  | -47 (-84, -13) | -11.1 (-18.5, -3.8) |
| Chungnam | 681 | -95 (-153, -36) | -12.3 (-18.4, -5.6) |  | -95 (-155, -40) | -12.3 (-18.9, -5.4) |
| Jeonbuk | 455 | -42 (-80, -2) | -8.4 (-15.2, -1.1) |  | -41 (-82, -4) | -8.3 (-15.6, -0.9) |
| Jeonnam | 452 | -23 (-61, 15) | -4.8 (-11.9, 2.9) |  | -22 (-62, 14) | -4.6 (-12.3, 3.2) |
| Gyungbuk | 659 | -72 (-127, -16) | -9.9 (-16.2, -3.1) |  | -69 (-126, -17) | -9.5 (-16.4, -2.4) |
| Gyungnam | 729 | -104 (-167, -43) | -12.5 (-18.6, -6.2) |  | -106 (-171, -48) | -12.7 (-19.3, -5.9) |
| Jeju | 174 | -23 (-40, -6) | -11.6 (-18.9, -4.0) |  | -23 (-41, -8) | -11.8 (-19.4, -4.2) |
| Korea | 11,513 | -1,185 (-2,042, -298) | -9.3 (-15.2, -3.1) |  | -1,196 (-1,900, -596) | -9.4 (-14.2, -4.8) |

eCI, empirical confidence interval ^a^We computed empirical confidence intervals (eCIs) at 95% by Monte Carlo simulation.

**Table S10.** Sensitivity analysis with different length of lag days of lag-response association in temperature basis term:
Total number, estimated excess (95% eCI^a^), and percent excess (95% eCI^a^) of suicide during period February 18–December 31, 2020
by region

|  | Total | Up to 5 lag days | |  | Up to 7 lag days | |  | Up to 10 lag days | |
| --- | --- | --- | --- | --- | --- | --- | --- | --- | --- |
|  |  | Excess | Percent excess (%) |  | Excess | Percent excess (%) |  | Excess | Percent excess (%) |
| Seoul | 1,902 | -88 (-213, 32) | -4.4 (-10.2, 1.6) |  | -80 (-216, 38) | -4.0 (-9.7, 2.5) |  | -70 (-195, 54) | -3.6 (-9.8, 3.0) |
| Busan | 803 | -102 (-167, -43) | -11.2 (-17.3, -4.9) |  | -107 (-179, -46) | -11.8 (-17.7, -4.9) |  | -112 (-180, -44) | -12.3 (-18.7, -5.4) |
| Daegu | 551 | -32 (-109, -21) | -10.1 (-16.7, -3.2) |  | -64 (-116, -20) | -10.4 (-16.9, -3.0) |  | -63 (-118, -17) | -10.3 (-17.4, -3.1) |
| Incheon | 664 | -72 (-129, -23) | -9.8 (-16.3, -3.0) |  | -74 (-135, -21) | -10.0 (-16.4, -3.7) |  | -73 (-129, -17) | -9.9 (-16.7, -2.5) |
| Gwangju | 286 | -37 (-63, -15) | -11.4 (-18.1, -4.5) |  | -37 (-65, -12) | -11.6 (-18.2, -4.3) |  | -38 (-65, -13) | -11.9 (-19.1, -4.2) |
| Daejeon | 340 | -37 (-69, -10) | -9.8 (-16.8, -2.3) |  | -38 (-72, -8) | -10.1 (-17.2, -2.8) |  | -35 (-68, -3) | -9.4 (-17.3, -1.2) |
| Ulsan | 266 | -32 (-58, -10) | -10.8 (-17.9, -3.5) |  | -33 (-61, -9) | -11.1 (-18.2, -3.9) |  | -33 (-59, -9) | -11.1 (-18.9, -3.1) |
| Gyunggi | 2,730 | -319 (-505, -142) | -10.5 (-15.7, -5.4) |  | -322 (-509, -146) | -10.5 (-15.7, -5.4) |  | -307 (-485, -129) | -10.1 (-15.5, -4.4) |
| Gangwon | 445 | -38 (-76, -4) | -7.8 (-14.8, -0.6) |  | -39 (-82, -2) | -8.0 (-15.1, -1.0) |  | -36 (-75, 4) | -7.5 (-15.0, 0.5) |
| Chungbuk | 376 | -45 (-79, -15) | -10.6 (-17.5, -3.5) |  | -47 (-84, -13) | -11.1 (-17.6, -3.6) |  | -46 (-80, -11) | -10.8 (-18.2, -2.8) |
| Chungnam | 681 | -92 (-152, -42) | -12.0 (-18.3, -5.5) |  | -96 (-159, -42) | -12.3 (-18.4, -5.4) |  | -98 (-164, -42) | -12.5 (-19.1, -5.8) |
| Jeonbuk | 455 | -44 (-85, -10) | -8.8 (-15.7, -1.7) |  | -43 (-88, -7) | -8.7 (-15.4, -0.7) |  | -43 (-88, -5) | -8.7 (-15.9, -1.2) |
| Jeonnam | 452 | -27 (-67, 6) | -5.7 (-13.0, 1.8) |  | -25 (-67, 12) | -5.3 (-12.5, 2.0) |  | -21 (-59, 18) | -4.4 (-12.3, 3.4) |
| Gyungbuk | 659 | -71 (-128, -23) | -9.7 (-16.3, -3.0) |  | -75 (-134, -23) | -10.2 (-16.4, -3.0) |  | -73 (-129, -17) | -9.9 (-16.8, -2.6) |
| Gyungnam | 729 | -99 (-161, -41) | -11.9 (-18.2, -5.7) |  | -103 (-170, -46) | -12.4 (-18.4, -5.4) |  | -105 (-168, -43) | -12.6 (-19.3, -5.6) |
| Jeju | 174 | -23 (-39, -9) | -11.5 (-18.4, -4.2) |  | -23 (-41, -7) | -11.8 (-18.8, -4.6) |  | -24 (-41, -7) | -12.0 (-19.6, -4.4) |
| Korea | 11,513 | -1,187 (-1,959, -558) | -9.3 (-14.5, -4.3) |  | -1,207 (-1,996, -522) | -9.5 (-14.4, -4.5) |  | -1,177 (-1,963, -407) | -9.3 (-15.0, -3.5) |

eCI, empirical confidence interval ^a^We computed empirical confidence intervals (eCIs) at 95% by Monte Carlo simulation.

**Table S11.** Sensitivity analysis with three knots in relative humidity spline term:
Total number, estimated excess (95% eCI^a^), and percent excess (95% eCI^a^) of
suicide during period February 18–December 31, 2020 by region

|  | Total | Excess | Percent excess (%) |
| --- | --- | --- | --- |
| Seoul | 1,902 | -87 (-216, 30) | -4.4 (-10.0, 1.3) |
| Busan | 803 | -99 (-167, -37) | -11.0 (-17.1, -5.1) |
| Daegu | 551 | -63 (-112, -21) | -10.2 (-16.8, -4.0) |
| Incheon | 664 | -77 (-132, -27) | -10.3 (-16.4, -4.5) |
| Gwangju | 286 | -35 (-61, -12) | -10.9 (-17.4, -4.7) |
| Daejeon | 340 | -39 (-71, -11) | -10.2 (-17.1, -3.7) |
| Ulsan | 266 | -31 (-56, -9) | -10.5 (-17.2, -3.9) |
| Gyunggi | 2,730 | -324 (-509, -154) | -10.6 (-15.8, -5.8) |
| Gangwon | 445 | -36 (-74, -1) | -7.6 (-14.3, -0.7) |
| Chungbuk | 376 | -45 (-80, -15) | -10.8 (-17.3, -4.4) |
| Chungnam | 681 | -90 (-151, -39) | -11.7 (-18.0, -6.0) |
| Jeonbuk | 455 | -43 (-83, -9) | -8.6 (-15.1, -2.4) |
| Jeonnam | 452 | -28 (-68, 6) | -5.9 (-12.8, 0.7) |
| Gyungbuk | 659 | -70 (-125, -21) | -9.6 (-15.8, -3.7) |
| Gyungnam | 729 | -99 (-162, -44) | -11.9 (-18.1, -6.1) |
| Jeju | 174 | -22 (-39, -8) | -11.3 (-17.9, -4.8) |
| Korea | 11,513 | -1,190 (-2,039, -431) | -9.4 (-15.0, -4.1) |

eCI, empirical confidence interval ^a^We computed empirical confidence intervals (eCIs) at 95% by Monte Carlo simulation.

**2. Fig. S1–2**

**
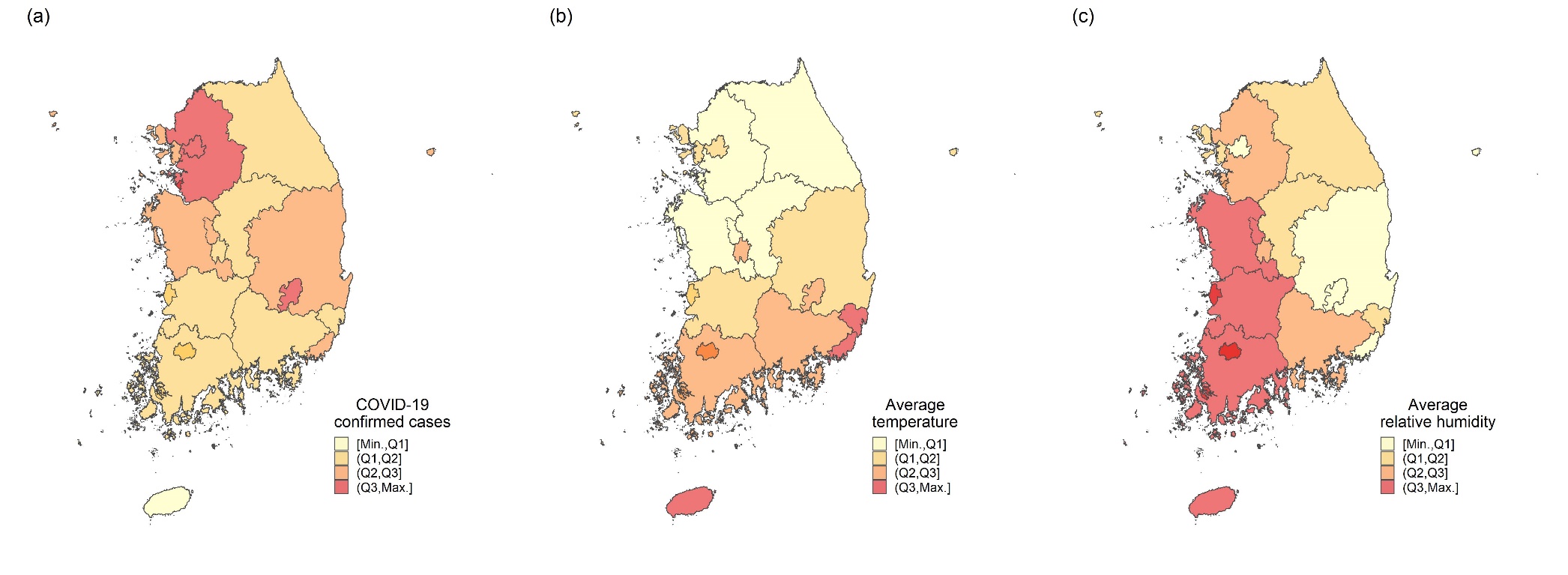
Fig. S1** Geographical distribution of the cumulative confirmed COVID-19 cases (a), average temperature (b), and average relative humidity (c) during the study period (January 1, 2017–December 31, 2020)


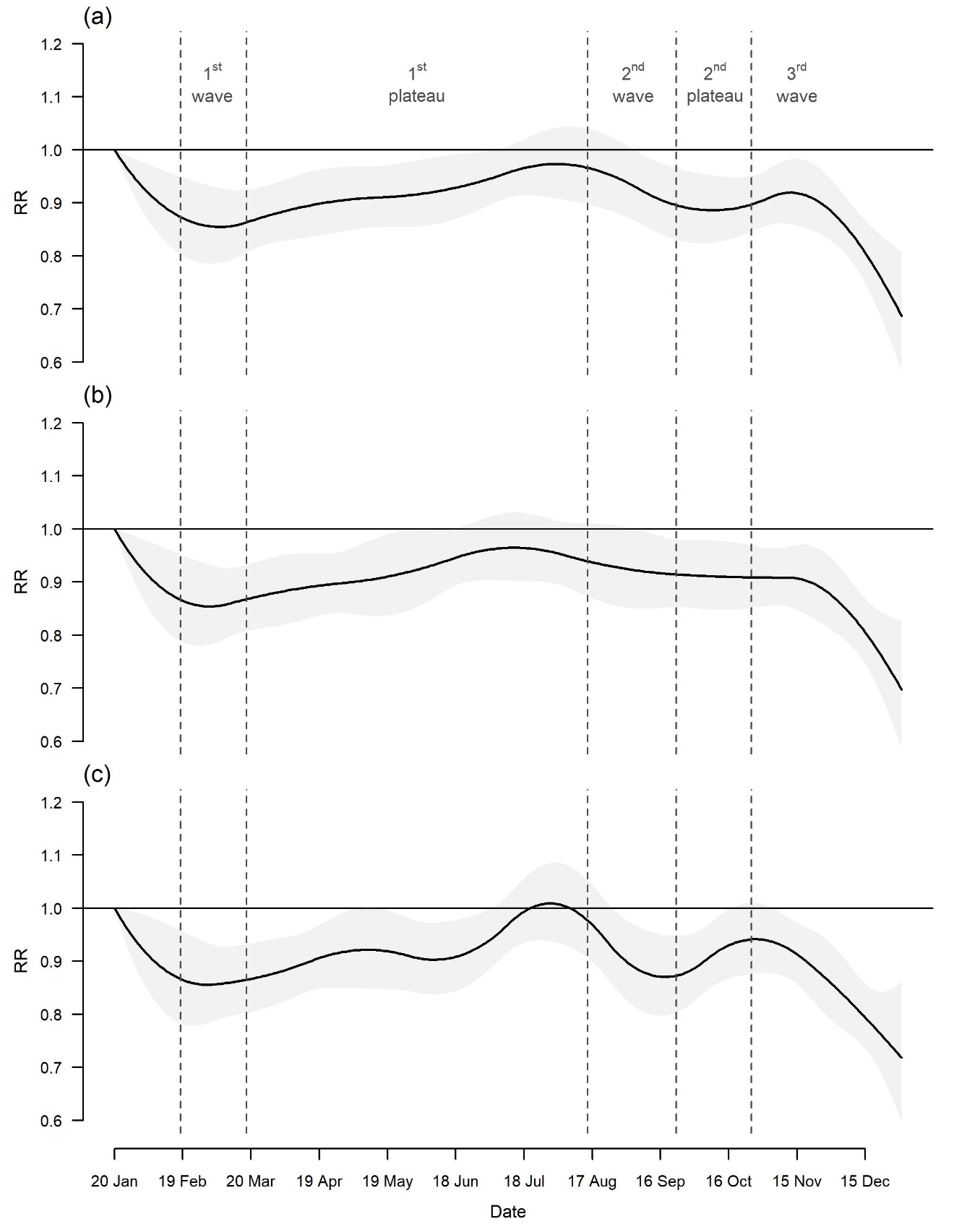
**Fig. S2** Sensitivity analysis with five (a), six (b), and seven (c) knots in the interrupted spline term: Temporal trend in excess risk (relative risk, RR) during the period January 20–December 31, 2020

**3. STROBE Statement**—checklist of items that should be included in reports of observational studies

|  | | Item No | Recommendation | | Page  No |
| --- | --- | --- | --- | --- | --- |
| **Title and abstract** | | 1 | (*a*) Indicate the study’s design with a commonly used term in the title or the abstract | | page 3 (Abstract) |
|  |  |  | (*b*) Provide in the abstract an informative and balanced summary of what was done and what was found | | page 3 (Abstract) |
| Introduction | | | | | |
| Background/rationale | | 2 | Explain the scientific background and rationale for the investigation being reported | | page 4 (Introduction) |
| Objectives | | 3 | State specific objectives, including any prespecified hypotheses | | page 5 (Introduction) |
| Methods | | | | | |
| Study design | | 4 | Present key elements of study design early in the paper | | page 6 (Methods) |
| Setting | | 5 | Describe the setting, locations, and relevant dates, including periods of recruitment, exposure, follow-up, and data collection | | page 6 (Methods) |
| Participants | | 6 | (*a*) *Cohort study*—Give the eligibility criteria, and the sources and methods of selection of participants. Describe methods of follow-up  *Case-control study*—Give the eligibility criteria, and the sources and methods of case ascertainment and control selection. Give the rationale for the choice of cases and controls  *Cross-sectional study*—Give the eligibility criteria, and the sources and methods of selection of participants | | page 6 (Methods) |
|  |  |  | (*b*) *Cohort study*—For matched studies, give matching criteria and number of exposed and unexposed  *Case-control study*—For matched studies, give matching criteria and the number of controls per case | | NA. |
| Variables | | 7 | Clearly define all outcomes, exposures, predictors, potential confounders, and effect modifiers. Give diagnostic criteria, if applicable | | page 6 (Methods) |
| Data sources/ measurement | | 8* | For each variable of interest, give sources of data and details of methods of assessment (measurement). Describe comparability of assessment methods if there is more than one group | | page 6 (Methods) |
| Bias | | 9 | Describe any efforts to address potential sources of bias | | page 9 (Methods –Sensitivity analysis) |
| Study size | | 10 | Explain how the study size was arrived at | | page 6-7 (Methods) |
| Quantitative variables | | 11 | Explain how quantitative variables were handled in the analyses. If applicable, describe which groupings were chosen and why | | page 7 (Methods) |
| Statistical methods | | 12 | (*a*) Describe all statistical methods, including those used to control for confounding | | page 7 (Methods – Statistical Analysis) |
|  |  |  | (*b*) Describe any methods used to examine subgroups and interactions | | page 9 (Methods – Sub-group analysis) |
|  |  |  | (*c*) Explain how missing data were addressed | | NA. |
|  |  |  | (*d*) *Cohort study*—If applicable, explain how loss to follow-up was addressed  *Case-control study*—If applicable, explain how matching of cases and controls was addressed  *Cross-sectional study*—If applicable, describe analytical methods taking account of sampling strategy | | NA. |
|  |  |  | (*e*) Describe any sensitivity analyses | | page 9 (Methods – Sensitivity analysis) |
| Results | | | | | |
| Participants | 13* | (a) Report numbers of individuals at each stage of study—eg numbers potentially eligible, examined for eligibility, confirmed eligible, included in the study, completing follow-up, and analysed | | page 10 (Results) | |
|  |  | (b) Give reasons for non-participation at each stage | | NA. | |
|  |  | (c) Consider use of a flow diagram | | - | |
| Descriptive data | 14* | (a) Give characteristics of study participants (eg demographic, clinical, social) and information on exposures and potential confounders | | page 10  (Results), & Table 1 | |
|  |  | (b) Indicate number of participants with missing data for each variable of interest | | NA. | |
|  |  | (c) *Cohort study*—Summarise follow-up time (eg, average and total amount) | | NA. | |
| Outcome data | 15* | *Cohort study*—Report numbers of outcome events or summary measures over time | | NA. | |
|  |  | *Case-control study—*Report numbers in each exposure category, or summary measures of exposure | | NA. | |
|  |  | *Cross-sectional study—*Report numbers of outcome events or summary measures | | page 10 (Results) | |
| Main results | 16 | (*a*) Give unadjusted estimates and, if applicable, confounder-adjusted estimates and their precision (eg, 95% confidence interval). Make clear which confounders were adjusted for and why they were included | | page 10 (Results), & Table 2 | |
|  |  | (*b*) Report category boundaries when continuous variables were categorized | | NA. | |
|  |  | (*c*) If relevant, consider translating estimates of relative risk into absolute risk for a meaningful time period | | NA. | |
| Other analyses | 17 | Report other analyses done—eg analyses of subgroups and interactions, and sensitivity analyses | | page 10-11 (Results), & Figure 2-3 | |
| Discussion | | | | | |
| Key results | 18 | Summarise key results with reference to study objectives | | page 11-12 (Discussion) | |
| Limitations | 19 | Discuss limitations of the study, taking into account sources of potential bias or imprecision. Discuss both direction and magnitude of any potential bias | | page 14 (Discussion) | |
| Interpretation | 20 | Give a cautious overall interpretation of results considering objectives, limitations, multiplicity of analyses, results from similar studies, and other relevant evidence | | page 11-12 (Discussion) | |
| Generalisability | 21 | Discuss the generalisability (external validity) of the study results | | page 12-13 (Discussion) | |
| Other information | | | | | |
| Funding | 22 | Give the source of funding and the role of the funders for the present study and, if applicable, for the original study on which the present article is based | | This work was supported by the National Research Foundation of Korea (BK21 Center for Integrative Response to Health Disasters, Graduate School of Public Health, Seoul National University) (NO.4199990514025). | |

**Note:** An Explanation and Elaboration article discusses each checklist item and gives methodological background and published examples of transparent reporting. The STROBE checklist is best used in conjunction with this article (freely available on the Web sites of PLoS Medicine at http://www.plosmedicine.org/, Annals of Internal Medicine at http://www.annals.org/, and Epidemiology at http://www.epidem.com/). Information on the STROBE Initiative is available at www.strobe-statement.org.

**4. R code for statistical analyses**

**[Two-stage interrupted time-series]**

################################################################################

# FIRST STAGE. Interrupted time-series analysis by city

### Load the packages

library(dlnm) ; library(tsModel) ; library(splines) ; library(pbs)

### Load the data

load("datamodel.Rda")

### Parameters

# The knots for the splines for modeling excess in post-period

nkpost <- 4

# The df for the cyclic spline for seasonality

dfseas <- 4

# Parameters of cross-basis for temperature

lagtmean <- 2

kpertmean <- c(25,50,75)

### Model formula

formula <- suicide_all ~ bpost + date + bseas + factor(wday(date)) + cbtmean + ns(rhum,2)

# List to store coef/vcov and convergence indicator

sidocode <- unique(datamodel$citycode)

stage1list <- vector("list",length(sidocode))

names(stage1list) <- unique(datamodel$city)

# Loop across city

for (i in seq(length(sidocode))) {

cat(unique(datamodel$city)[i],"")

dd <- subset(datamodel, citycode == sidocode[i])

# Define basis functions for post-period, seasonality, temperature

# N.B. Use onebasis to simplify predictions and plotting

kpost <- equalknots(dd$tspost, nkpost)

bpost <- onebasis(dd$tspost, fun="bs", degree=2, knots=kpost)

kseas <- equalknots(yday(dd$date), dfseas)

bseas <- onebasis(yday(dd$date), fun="pbs", knots=kseas)

cbtmean <- crossbasis(dd$tmean, lag=lagtmean,

argvar=list(fun="bs", degree=2, knots=quantile(dd$tmean, kpertmean/100)),

arglag=list(fun="strata",breaks=1))

# Run the model

# N.B. Preserve missing to compute residuals later

mod <- glm(formula, data=dd, family=quasipoisson, na.action="na.exclude")

# Save the results: coef/vcov, residuals, overdispersion

loglik <- sum(dpois(mod$y,mod$fitted.values,log=TRUE))

disp <- sum(residuals(mod,type="pearson")^2,na.rm=T)/mod$df.residual

stage1list[[i]] <- list(coef=coef(mod), vcov=vcov(mod), dispersion=disp,

residuals=residuals(mod, type="deviance"))

}

################################################################################

# SECOND-STAGE. Meta-analysis

library(mixmeta)

# Multivariate meta-analysis of coefficients of post-period excess

indpost <- grep("bpost", names(stage1list[[1]]$coef))

coefpost <- t(sapply(stage1list, function(x) x$coef[indpost]))

Scov <- lapply(stage1list, function(x) x$vcov[indpost,indpost])

metapost <- mixmeta(coefpost, Scov)

bluppost <- blup(metapost, vcov=T)

**[Quantification of excess suicide]**

################################################################################

# COMPUTE EXCESS MORTALITY

# Number of resampling iterations for empirical CI

nsim <- 1000

# Start and end day for post-period and covid period

startdate <- dmy(20012020) #2020-01-20

coviddate <- dmy(18022020) #2020-02-18

enddate <- dmy(31122020) #2020-12-31

# Week periods for Jan-Dec

seqpost <- seq(startdate,enddate,1)

cutdate <- unique(c(startdate-1,seqpost[seqpost %in% tapply(seqpost,week(seqpost),last)]))

labperiod1 <- sapply(seq(length(cutdate)-1), function(i)

paste(paste0(day(cutdate[i]+1), month(cutdate[i]+1,lab=T)),

paste0(day(cutdate[i+1]), month(cutdate[i+1],lab=T)), sep="-"))

labperiod2 <- paste("Week",unique(week(seqpost)))

# Define periods

seqperiod <- cut(unique(datamodel$tspost),cutdate-startdate,labels=labperiod2)

# Redefine basis

kpost <- equalknots(datamodel$tspost,nkpost)

bpost <- onebasis(unique(datamodel$tspost),fun="bs",degree=2,knots=kpost)

# Define array to store the excess deaths by sido, peroid, resampling

excitysim <- array(NA,dim=c(length(sidocode),length(labperiod1)+1,nsim+1),

dimnames=list(unique(datamodel$city),c("18Feb-31Dec",labperiod1),

c("est",paste0("sim",seq(nsim)))))

excity_daily <- array(NA,dim=c(347,17,nsim+1),

dimnames=list(seq(startdate,enddate,1),c(unique(datamodel$city),"korea"),c("an",paste0("sim",seq(nsim)))))

# Loop across sido

library(MASS)

for (i in seq(length(sidocode))) {

cat(unique(datamodel$city)[i],"")

# Retrieve coef/vcov and total deaths

coef <- bluppost[[i]]$blup

vcov <- bluppost[[i]]$vcov

death <- subset(datamodel,citycode==sidocode[i] & date>=startdate)$suicide_all

# Compute attributable number (excess), and store the sum by sido

an <- (1-exp(-bpost%*%coef))*death

indcovid <- seqpost>=coviddate

excitysim[i,1,"est"] <- sum(an[indcovid])

excitysim[i,-1,"est"] <- tapply(an,seqperiod,sum)

excity_daily[,i,1] <- an

# Sample coef assuming a MVN dist.

set.seed(19970302)

coefsim <- mvrnorm(nsim,coef,vcov)

# Loop across interactions and do as above with resampling coef

for (s in seq(nsim)) {

an <- (1-exp(-bpost%*%coefsim[s,]))*death

excitysim[i,1,s+1] <- sum(an[indcovid])

excitysim[i,-1,s+1] <- tapply(an,seqperiod,sum)

excity_daily[,i,s+1] <- an

}

}

# Collapse in full country

exkoreasim <- apply(excitysim,2:3,sum)

for (i in 1:1001) {

excity_daily[,17,i] <- rowSums(excity_daily[,-17,i])

}
